# Objective Assessment of the Utility of Chromoendoscopy with a Support Vector Machine

**DOI:** 10.1007/s12029-018-0083-6

**Published:** 2018-03-05

**Authors:** Ryo Ogawa, Jun Nishikawa, Eizaburo Hideura, Atsushi Goto, Yurika Koto, Shunsuke Ito, Madoka Unno, Yuko Yamaoka, Ryo Kawasato, Shinichi Hashimoto, Takeshi Okamoto, Hiroyuki Ogihara, Yoshihiko Hamamoto, Isao Sakaida

**Affiliations:** 1 Department of Gastroenterology and Hepatology, Yamaguchi University Graduate School of Medicine, 1-1-1 Minamikogushi, Ube, Japan; 2 Department of Laboratory Science, Yamaguchi University Graduate School of Medicine, 1-1-1 Minamikogushi, Ube, Japan; 3 Department of Biomolecular Engineering Applied Molecular Bioscience, Yamaguchi University Graduate School of Medicine, 2-16-1 Tokiwadai, Ube, Japan; 4 Division of Electrical, Electronic and Information Engineering, Yamaguchi University Graduate School of Sciences and Technology for Innovation, 2-16-1 Tokiwadai, Ube, Japan

**Keywords:** Gastric cancer, Diagnosis, Support vector machine, Chromoendoscopy, Acetic acid, Indigo carmine

## Abstract

**Purpose:**

The utility of chromoendoscopy for early gastric cancer (GC) was determined by machine learning using data of color differences.

**Methods:**

Eighteen histopathologically confirmed early GC lesions were examined. We prepared images from white light endoscopy (WL), indigo carmine (Indigo), and acetic acid-indigo carmine chromoendoscopy (AIM). A border between cancerous and non-cancerous areas on endoscopic images was established from post-treatment pathological findings, and 2000 pixels with equivalent luminance values were randomly extracted from each image of cancerous and non-cancerous areas. Each pixel was represented as a three-dimensional vector with RGB values and defined as a sample. We evaluated the Mahalanobis distance using RGB values, indicative of color differences between cancerous and non-cancerous areas. We then conducted diagnosis test using a support vector machine (SVM) for each image. SVM was trained using the 100 training samples per class and determined which area each of 1900 test samples per class came from.

**Results:**

The means of the Mahalanobis distances for WL, Indigo, and AIM were 1.52, 1.32, and 2.53, respectively and there were no significant differences in the three modalities. Diagnosability per endoscopy technique was assessed using the F1 measure. The means of F1 measures for WL, Indigo, and AIM were 0.636, 0.618, and 0.687, respectively. AIM images were better than WL and Indigo images for the diagnosis of GC.

**Conclusion:**

Objective assessment by SVM found AIM to be suitable for diagnosis of early GC based on color differences.

## Introduction

The detectability of early gastric cancers has improved with the use of high-resolution video endoscopy and chromoendoscopy [1–3]. Magnifying endoscopy combined with image-enhanced endoscopy such as narrow-band imaging has also been reported to improve the qualitative diagnosability of gastric cancers [4–6]. However, previous assessment of the diagnosability of early gastric cancer using endoscopy was based on the endoscopist’s subjective judgment and thereby included some problems in its objectivity [7, 8]. Therefore, we used the three-dimensional vectors with RGB value, indicative of color differences between cancerous and non-cancerous areas. Then, objective assessment of the diagnosability per endoscopy technique was conducted by its discriminator generated following training of a support vector machine (SVM) [9].

Endoscopic diagnosis of early gastric cancer has carried out based on distinct differences in the surface color and properties between the lesion and the surrounding tissues. White light endoscopy (WL) is used for the screening of gastric cancer. Indigo carmine chromoendoscopy (Indigo) is simple and easy and is also thought to be useful for the diagnosis of cancer [7, 8]. Acetic acid-indigo carmine chromoendoscopy (AIM) is a method that is based on the difference in the level of mucus, which may be due to a defense reaction of the gastric mucosa against acetic acid, between cancerous and non-cancerous areas. It has been reported that the diagnosability of gastric cancer is high using AIM [10, 11].

There have been remarkable advances in the artificial intelligence field recently, and the application of this technology to the medical field is expected [12, 13]. However, there are few cases of its application to the endoscopic field except for the detection of dysplasia in patients with Barrett’s esophagus and diagnosis via capsule endoscopy [14, 15]. In the present study, by using a SVM, which is one of machine learning techniques available for high-precision discrimination, the diagnosability of gastric cancer using different endoscopy techniques was compared based on the color difference between the cancerous and non-cancerous areas.

## Materials and Methods

### Subjects

Eighteen patients underwent upper endoscopy and endoscopic resection or surgical resection at the Yamaguchi University Hospital, and 18 lesions that were diagnosed histopathologically as early gastric cancer were examined. Our 18 subjects were patients who were undergone endoscopic examination by WL, Indigo, and AIM from June 2014 to August 2016. In these cases, the images by WL, Indigo, and AIM were recorded as similar views. Clinicopathological features of cases of the early gastric carcinomas were shown in Table 1.

**Table 1:** Clinicopathological features of the early gastric carcinomas

Age	Mean	71.2
Sex	Male	13
	Female	5
Location	Upper	2
	Middle	5
	Lower	10
	Residual stomach	1
Lesion diameter	Mean	25
	< 20 mm	6
	≧ 20 mm ≧ 20 mm	12
Macroscopic type	Elevated type	6
	Depressed type	12
Color	Reddish	11
	Normal-colored	2
	Discolored	5
Tumor differentiation	Differentiated	17
	Undifferentiated	1
Invasion depth	Mucosal layer	13
	Submucosal layer	5
Coexistence of the ulcer	Positive	1
	Negative	17

This study was conducted after obtaining the approval of the ethics committee of the Yamaguchi University Hospital. All the 18 patients provided their informed consent before resection for images to be acquired.

### Image Acquisition

**Fig. 1:**
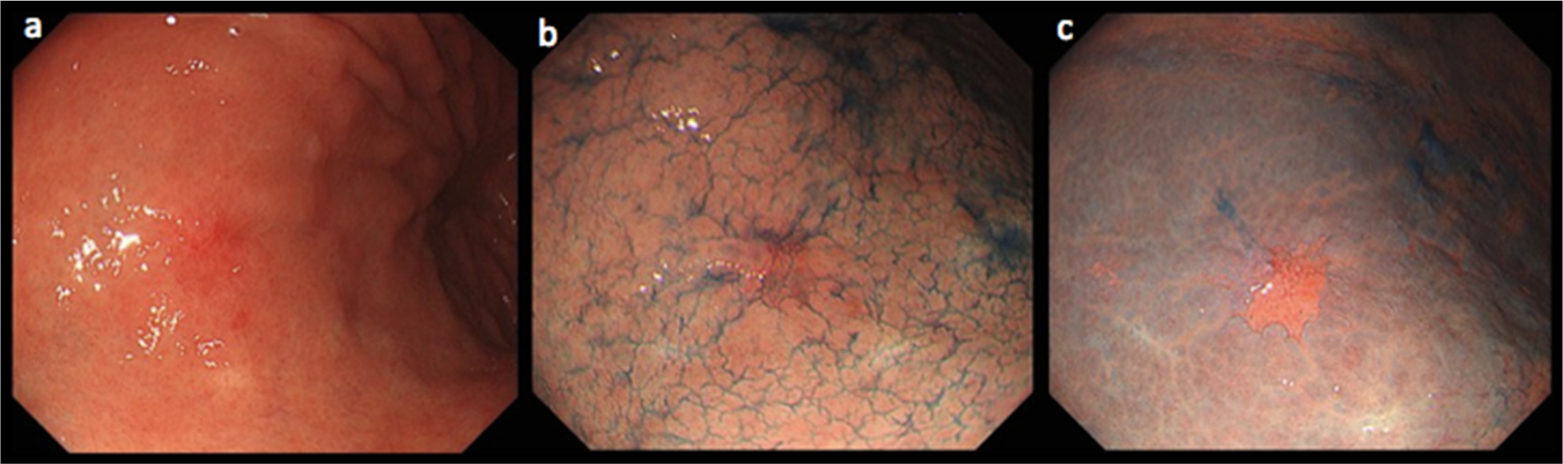
Representative images of early gastric carcinoma from white light endoscopy (**a**), indigo carmine chromoendoscopy (**b**), and acetic acid-indigo carmine chromoendoscopy (**c**)

The endoscope and the image processor used in our study were a GIF-H260Z and an EVIS LUCERA ELITE, respectively (both from Olympus, Tokyo, Japan). White light endoscopy was performed, and then 0.2% indigo carmine was dispersed. Following this, a solution consisting of 0.2% indigo carmine plus 2.1% acetic acid was dispersed, and the images were acquired 12 s later. The images of the same lesions were thus obtained in order through WL, Indigo, and AIM (Fig. 1).

### Determination of Tumor Boundary and Extraction of RGB Data

**Fig. 2:**
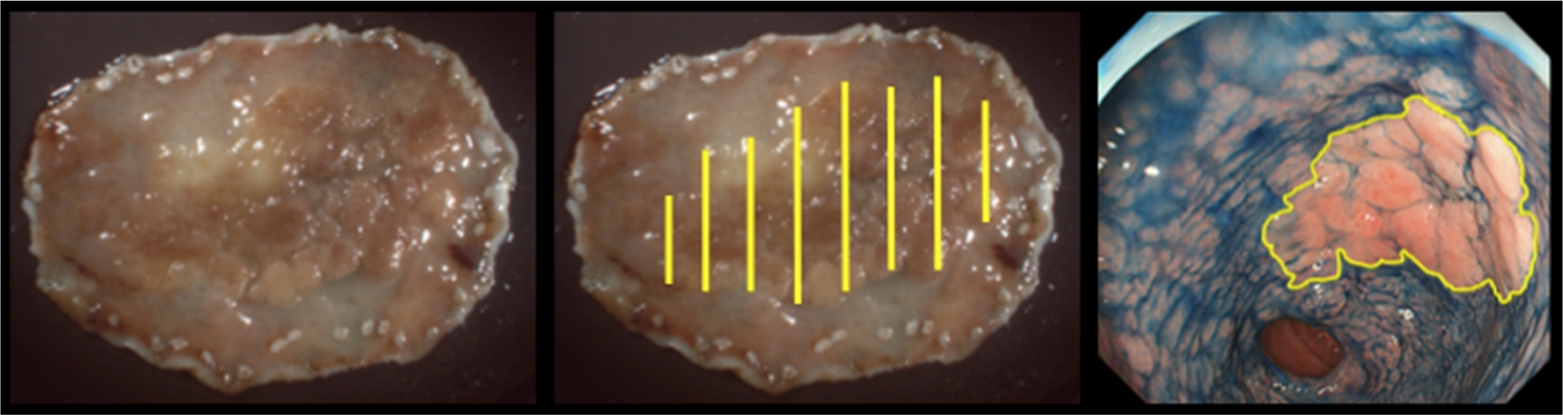
Determination of the tumor boundary. Whole specimens were obtained by endoscopic submucosal dissection or surgery. A pathologist and endoscopist made the pathological diagnosis, and then, the endoscopist made a stereoscopic microscopic diagnosis based on the pathological findings. Finally, the endoscopist created images by matching the cancerous area with the endoscopic image

A total of 54 still images of the 18 lesions were evaluated. A specialist certified by the Japan Gastroenterological Endoscopy Society (JN) examined the endoscopic images along with the corresponding macroscopic and histopathology findings and then circled a cancerous area on the individual images (Fig. 2).

**Fig. 3:**
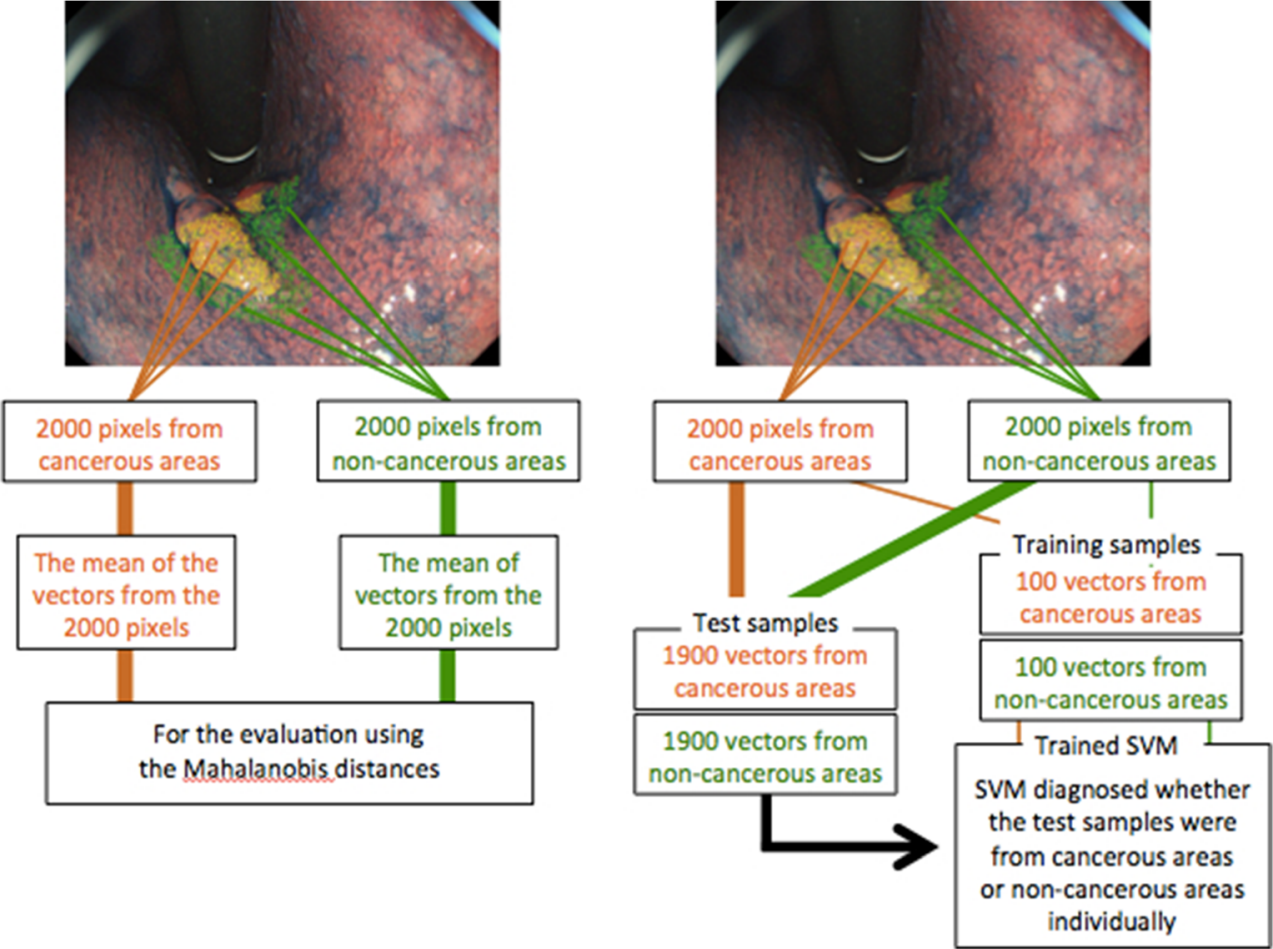
Extraction of RGB data. Two thousand pixel samples with equivalent intensity levels were randomly extracted from each image of cancerous and non-cancerous areas (4000 pixels in total). In the Mahalanobis distance method, the mean of RGB three-dimensional vectors from 4000 pixels was used for evaluation. In the SVM method, one hundred RGB three-dimensional vectors from the cancerous areas and one hundred vectors from the non-cancerous areas were used for training. The trained SVM diagnosed whether remaining 3800 RGB three-dimensional vectors were from cancerous or non-cancerous lesion individually

Pixels were extracted randomly from both the cancerous and non-cancerous areas on the images. To exclude the difference in the luminance value of the targeted pixels, the luminance value of each pixel was calculated from the extracted RGB values [16]. Then, 2000 pixels with equivalent luminance values were chosen from each image of the cancerous and non-cancerous areas (4000 pixels in total) obtained using the three endoscopy techniques. Following this, a pixel represented as a three-dimensional vector with RGB values was defined as a sample (Fig. 3).

### The Mahalanobis Distance

The mean vectors (*μ*_1_) and covariance matrix (*Σ*_1_) were estimated using 2000 samples in the cancerous area, and similarly, the mean vectors (*μ*_2_) and covariance matrix (*Σ*_2_) were estimated using 2000 samples in the non-cancerous area on the image (Fig. 3). The Mahalanobis distances, indicative of color differences between cancerous and non-cancerous areas, were obtained for WL, Indigo, and AIM, respectively [17]. Differences in the mean of Mahalanobis distances between WL and Indigo, WL and AIM, and Indigo and AIM were calculated respectively for each of the 18 lesions.

### Machine Learning and F1 Measure

One hundred samples from the cancerous areas and 100 samples from the non-cancerous areas were used for training, and the remaining 3800 samples (1900 samples from the cancerous and 1900 samples from the non-cancerous areas) were used for testing (Fig. 3). The LIBSVM (Library for Support Vector Machines) [18] was trained using the training samples. Then, the SVM diagnosed whether the test samples were from cancerous areas or non-cancerous areas. Each diagnosability per endoscopy technique was assessed using the F1 measure, which was calculated as the harmonic mean of the sensitivity (reproducible rate) and the positive predictive value (precision), and this measure was used as the indicator of diagnosability [19]. The mean of the F1 measures in each of the 18 lesions was statistically compared for each endoscopy technique.

### Subjective Assessment of the Images

Fifty-four still images with white light (WL), Indigo, and AIM of the 18 lesions were randomly sorted to prevent the influence of display order. Five endoscopists completely blinded to patient information including the pathological diagnosis and endoscopic images evaluated the 54 still images to determine whether gastric cancer could be diagnosed. Re-examination of previously examined images was strictly prohibited. The mean of the ratio of the images that the endoscopists could diagnose gastric cancer was statistically compared for each endoscopy technique.

## Results

**Fig. 4:**
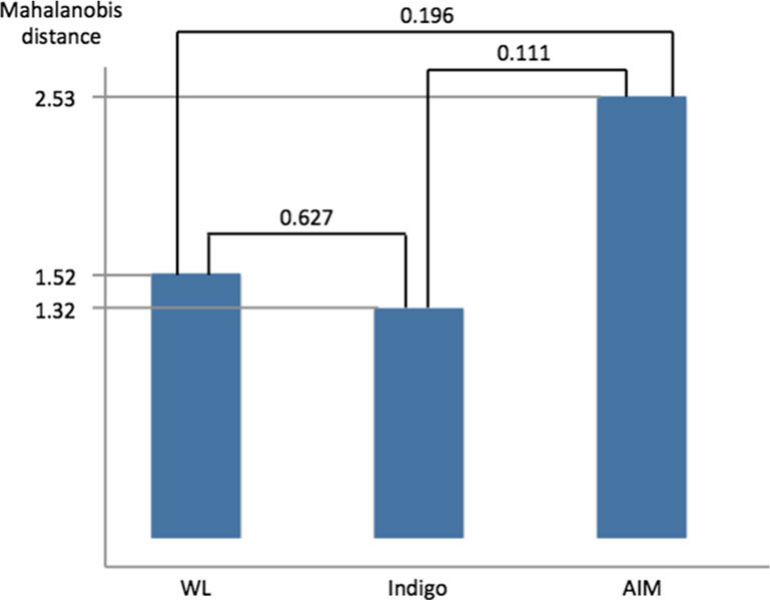
The means of the Mahalanobis distances with the white light (WL) endoscopy, indigo carmine (Indigo), and acetic acid-indigo carmine (AIM) chromoendoscopy were 1.52, 1.32, and 2.53 respectively. The AIM images tend to be superior to the WL and Indigo images. There are no significant differences

The means of the Mahalanobis distances with WL, Indigo, and AIM were 1.52, 1.32, and 2.53 respectively. The AIM images tend to be superior in color difference to WL and Indigo images. There were no significant differences in the three modalities (Fig. 4).

**Fig. 5:**
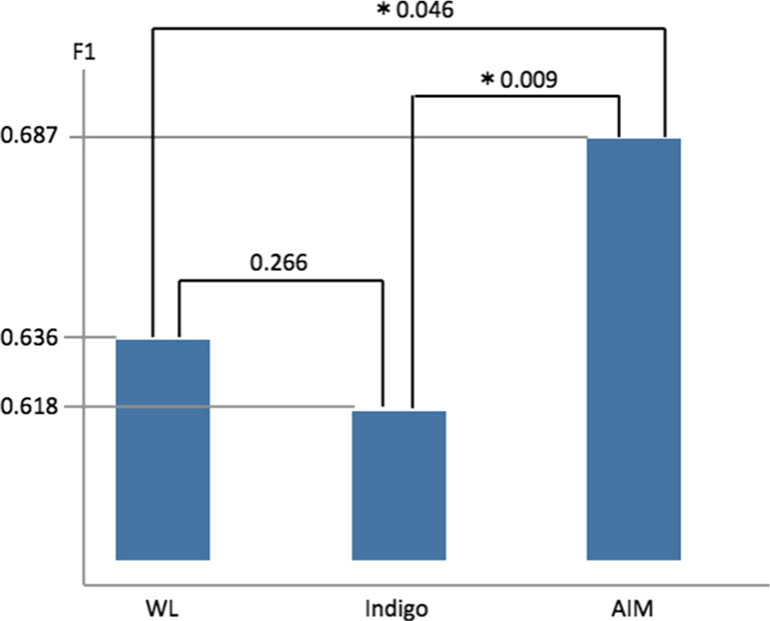
F1 measure of each endoscopic technique. F1 scores of the white light (WL) endoscopy, indigo carmine (Indigo), and acetic acid-indigo carmine (AIM) chromoendoscopy were 0.636, 0.618, and 0.687. The AIM images were superior to the WL and Indigo images

When we compared each diagnosability by using SVM, the means of the F1 measures with WL, Indigo, and AIM were 0.636, 0.618, and 0.687, respectively. These results indicated that the images from the AIM were suitable for the diagnosis of gastric cancer based on color differences (Fig. 5).

**Fig. 6:**
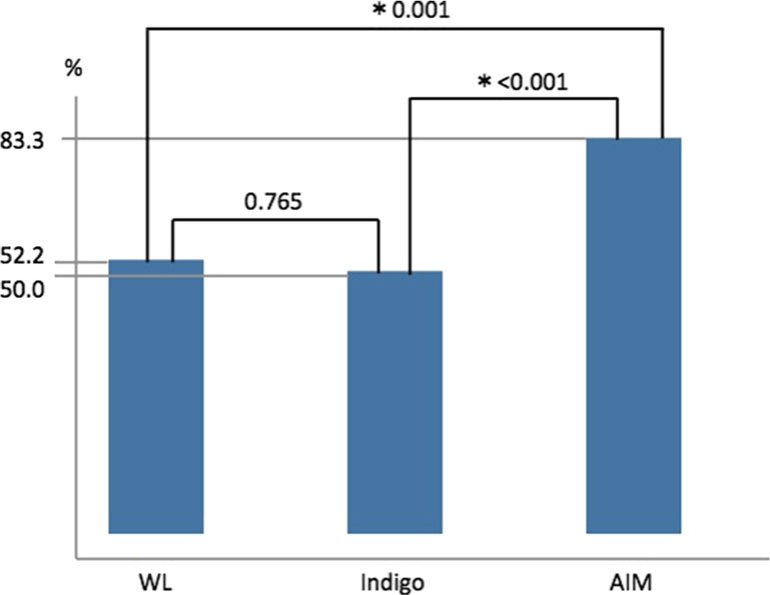
The mean of the ratio of the images that the endoscopists could diagnose gastric cancer. The mean rates of diagnosability for WL, Indigo, and AIM were 50.0, 52.2, and 83.3%, respectively

From the subjective assessment, the mean rates that the endoscopists could diagnose gastric cancer for WL, Indigo, and AIM were 50.0, 52.2, and 83.3%, respectively (Fig. 6). Compared with the results for the WL and Indigo images, the mean ratio of diagnosability was significantly higher for the AIM images.

## Discussion

The usefulness of indigo carmine and AIM was generally assessed based on the endoscopist’s subjective assessment [7, 8]. Objective tests of each technique in the endoscopy field are rarely performed. The Mahalanobis distances and machine learning using the RGB values of the pixels extracted randomly from the endoscopic image were used to objectively assess the endoscopic technique based on color differences. We previously reported the diagnostic ability of optical enhancement system in early gastric cancer demarcation with the same method [16]. The means of the Mahalanobis distances with the three modalities were not significantly different while SVM worked well for discrimination of the utility of the three techniques.

Recently, machine learning has attracted attention as support for diagnostic tools in the medical field [12, 13]. The SVM used in our study is classified as a data-knowledge integration artificial intelligence and has excellent pattern discriminability. By setting a boundary line where the training sample can be distinguished at the maximum, the SVM can identify two classes with high precision [20, 21]. The F1 measure, which was used as the indicator of diagnosability, is considered an accurate indicator focusing not only on the sensitivity but also on the positive predictive value [19]. Van der Sommen et al. showed the usefulness of the SVM and the F1 measure for evaluating the endoscopic image of Barrett’s esophagus [14].

Endoscopic diagnosis of early gastric cancer has carried out based on distinct differences in the surface color and properties between the lesion and the surrounding tissues. The images obtained from AIM were the most suitable for diagnosis of gastric cancer based on color differences compared with the images from WL and Indigo. Kawahara et al. reported that the outcome of endoscopic resection was good in lesions in which the gastric tumor margin was determined using AIM [10, 11]. The usefulness of this type of chromoendoscopy might be based on the enhanced color difference.

When a classifier designed by machine learning is used for the endoscopic diagnosis of gastric cancer, it is desirable to use a versatile system for various lesions. However, in a previous examination, it was found that the color of the cancerous areas in each lesion or the gastric mucosa of the non-cancerous areas was different on the endoscopic images of each patient. The individual variation results in the difficulty of diagnosis. Consequently, it is necessary to examine the color differences between cancerous areas and non-cancerous areas as the reference in individual patients [22].

As limitations of this study, it was performed with a small number of patients in a single institution. In addition, gastric cancer with differentiated tumors, for which endoscopic treatment is indicated, accounted for most of the cases. In the future, we plan to perform another study with more patients from multiple institutions and with various histologic types of gastric cancer.

## Conclusion

Objective assessment with a SVM showed AIM to be suitable technique for the diagnosis of early gastric cancer based on variances in color differences in the endoscopic images.

## References

[1] Kaise M, Kato M, Tajiri H (2010). High-definition endoscopy and magnifying endoscopy combined with narrow band imaging in gastric cancer. Gastroenterology Clinics of North America.

[2] Sugano K (2015). Detection and management of early gastric cancer. Curr Treat Options Gastroenterol.

[3] Lambert R, Saito H, Saito Y (2007). High-resolution endoscopy and early gastrointestinal cancer...dawn in the East. Endoscopy.

[4] Ezoe Y, Muto M, Uedo N, Doyama H, Yao K, Oda I, Kaneko K, Kawahara Y, Yokoi C, Sugiura Y, Ishikawa H, Takeuchi Y, Kaneko Y, Saito Y (2011). Magnifying narrowband imaging is more accurate than conventional white-light imaging in diagnosis of gastric mucosal cancer. Gastroenterology.

[5] Yao K, Anagnostopoulos GK, Ragunath K (2009). Magnifying endoscopy for diagnosing and delineating early gastric cancer. Endoscopy.

[6] Sumie H, Sumie S, Nakahara K, Watanabe Y, Matsuo K, Mukasa M, Sakai T, Yoshida H, Sata M (2014). Usefulness of magnifying endoscopy with narrow-band imaging for diagnosis of depressed gastric lesions. Mol Clin Oncol.

[7] Szalóki T (2002). Indigo carmine contrast staining in combination with high resolution electronic endoscopy. Orv Hetil.

[8] Dinis-Ribeiro M (2006). Chromoendoscopy for early diagnosis of gastric cancer. Eur J Gastroenterol Hepatol.

[9] Vladimir NV (1995) The nature of statistical learning theory. Springer-Verlag, New York.

[10] Numata N, Oka S, Tanaka S, Yoshifuku Y, Miwata T, Sanomura Y, Arihiro K, Shimamoto F, Chayama K (2016). Useful condition of chromoendoscopy with indigo carmine and acetic acid for identifying a demarcation line prior to endoscopic submucosal dissection for early gastric cancer. BMC Gastroenterol.

[11] Kawahara Y, Takenaka R, Okada H, Kawano S, Inoue M, Tsuzuki T, Tanioka D, Hori K, Yamamoto K (2009). Novel chromoendoscopic method using an acetic acid-indigo carmine mixture for diagnostic accuracy in delineating the margin of early gastric cancers. Dig Endosc.

[12] Nishio M, Nagashima C (2017). Computer-aided diagnosis for lung cancer: usefulness of nodule heterogeneity. Acad Radiol.

[13] Chang CC, Chen HH, Chang YC, Yang MY, Lo CM, Ko WC, Lee YF, Liu KL, Chang RF (2017). Computer-aided diagnosis of liver tumors on computed tomography images. Comput Methods Prog Biomed.

[14] Van der Sommen F, Zinger S, Curvers WL, Bisschops R, Pech O, Weusten BL, Bergman JJ, De with PH, Schoon EJ (2016). Computer-aided detection of early neoplastic lesions in Barrett’s esophagus. Endoscopy.

[15] Kodogiannis VS, Boulougoura M, Lygouras JN, Petrounias I (2005). A neuro-fuzzy-based system for detecting abnormal patterns in wireless-capsule endoscopic images. Neurocomputing.

[16] Nagao M, Nishikawa J, Ogawa R, Sasaki S, Nakamura M, Nishimura J, Goto A, Hashimoto S, Okamoto T, Suenaga M, Hamamoto Y, Sakaida I. Evaluation of the diagnostic ability of optical enhancement system in early gastric cancer demarcation. Gastroenterol Res Pract. 2016;2016:2439621. 10.1155/2016/2439621. 10.1155/2016/2439621 PMC5059582 27774101

[17] Mahalanobis PC (1936) On the generalized distance in statistics. In: Proc Nat Inst Sciences, India, pp 49–55.

[18] Chang CC, Lin CJ (2011). LIBSVM: a library for support vector machines. ACM Trans Intell Syst Technol.

[19] Powers DMW (2011). Evaluation: from precision, recall and f-measure to ROC, informedness, markedness & correlation. Journal of Machine Learning Technology.

[20] Hassanpour S, Langlotz CP, Amrhein TJ, Befera NT, Lungren MP (2017). Performance of a machine learning classifier of knee MRI reports in two large academic radiology practices: a tool to estimate diagnostic yield. AJR Am J Roentgenol.

[21] Li Y, Zhang T (2017). Deep neural mapping support vector machines. Neural Netw.

[22] Kiyotoki S, Nishikawa J, Okamoto T, Hamabe K, Saito M, Goto A, Fujita Y, Hamamoto Y, Takeuchi Y, Satori S, Sakaida I (2013). New method for detection of gastric cancer by hyperspectral imaging: a pilot study. J Biomed Opt.

